# Comprehensive Genomic Analysis of Marine Strain *Streptomyces* sp. 891, an Excellent Producer of Chrysomycin A with Therapeutic Potential

**DOI:** 10.3390/md20050287

**Published:** 2022-04-24

**Authors:** Xu Hu, Yuqi Tang, Yuanyuan Liu, Xinwei Pei, Ziwei Huang, Fuhang Song, Huawei Zhang

**Affiliations:** 1 School of Pharmaceutical Sciences, Zhejiang University of Technology, Hangzhou 310014, China; hu_xu2018@hznu.edu.cn (X.H.); t17670659912@163.com (Y.T.); liuyuan_0507@163.com (Y.L.); peixinwei350@163.com (X.P.); hzw15879152506@163.com (Z.H.); 2 College of Life and Environmental Sciences, Hangzhou Normal University, Hangzhou 311121, China; 3 School of Light Industry, Beijing Technology and Business University, Beijing 100048, China; songfuhang@btbu.edu.cn

**Keywords:** marine strain, *Streptomyces*, whole-genome, gene mining, chrysomycin A

## Abstract

Chrysomycin A is one of the most promising therapeutic candidates for treating infections caused by multidrug-resistant Gram-positive bacteria. By hybridizing next-step generation (Illumina) and third-generation (PacBio) sequencing technologies, a high-quality chromosome-level genome together with a plasmid was firstly assembled for chrysomycin A-producing marine strain 891. Phylogenetic analysis of the 16*S* rRNA gene and genome sequences revealed that this strain unambiguously belonged to the genus *Streptomyces*, and its genomic features and functional genes were comprehensively analyzed and annotated. AntiSMASH analysis of this strain unveiled one key biosynthetic gene cluster, T2PKS, responsible for the biosynthesis of chrysomycin, the biosynthesis pathway of which was putatively proposed. These findings definitely shed light on further investigation for construction of a robust industrial strain with high-yield chrysomycin A production using genetic engineering techniques and combinatorial biology approaches.

## 1. Introduction

Terrestrial and marine *Streptomyces* strains play an important role in new drug discovery and development since they harbor a huge number of secondary metabolite biosynthetic gene clusters (BGCs) to make a diverse array of bioactive substances with therapeutic potential [1,2]. *Streptomyces* bacteria are known to produce numerous natural products, most of which are clinically useful compounds with antibacterial, antifungal, anticancer, immunosuppressive, and other properties. It is well known that daptomycin is a cyclic lipopeptide antibiotic derived from the organism *Streptomyces roseosporus* [3], rapamycin is used as immunosuppressive metabolite from actinomycete species [4], and avermectins are a series of drugs and pesticides from *Streptomyces avermitilis* [5]. However, the misuse and overuse of existing antibiotics in human medical practice is liable to cause seriously antimicrobial resistance, which has emerged as one of the leading public health threats around the world [6]. Therefore, there is an urgent need to constantly search for new drug candidates to alleviate this deteriorative tendency. In the post-genomic era, the accumulation of genomic and transcriptomic information is accelerating and has revealed that the metabolic capacity of virtually all organisms is vastly underappreciated. Genome mining is one of the effective strategies to increase the discovery rate and facilitate the characterization of novel compounds and their biosynthetic pathways [7].

**Figure 1:**
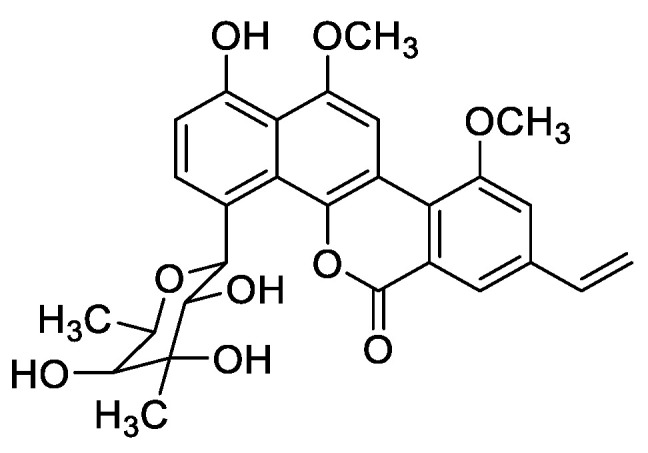
Chemical structure of chrysomycin A.

Chrysomycin analogs are one group of glycosides with a benzonaphthopyranone structure obtained from several *Streptomyces* strains [8,9], which display a broad spectrum of biological properties, including anti-phage, anti-bacterial, and cytotoxic activities [10,11,12,13]. Especially, chrysomycin A (Figure 1) showed a potent inhibitory effect on multidrug-resistant (MDR) and extensively drug-resistant (XDR) *Mycobacterium tuberculosis*, methicillin-resistant *Staphylococcus aureus* (MRSA), and vancomycin-resistant *Enterococcus* (VRE) [13,14,15,16]. Chrysomycin A was originally isolated from strain *Streptomyces* A-419 in 1955 as a mixture with chrysomycin B [10]. Strain 891 was derived from sediments of the South China Sea and found to produce chrysomycin A under optimal conditions with the highest yield (3600 mg/L) among all documented strains (Table S1) [17]. In order to decipher and characterize genomic features of this marine strain, whole genome sequencing and analysis were well conducted in this work, and the BGC responsible for the synthesis of chrysomycin was extensively analyzed. To our knowledge, it is the first report on bioinformative analysis of a chrysomycin-producing *Streptomyces* strain.

## 2. Results

### 2.1. Morphology, Classification, and Phylogenetic Analysis of Strain 891

**Figure 2:**
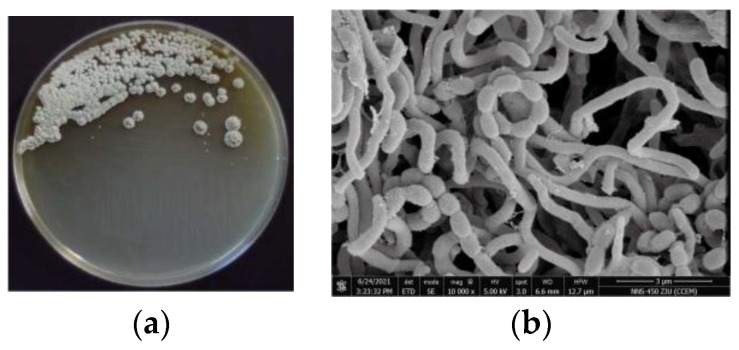
Colony (**a**) and microscopic (**b**) morphology of marine strain 891.

**Figure 3:**
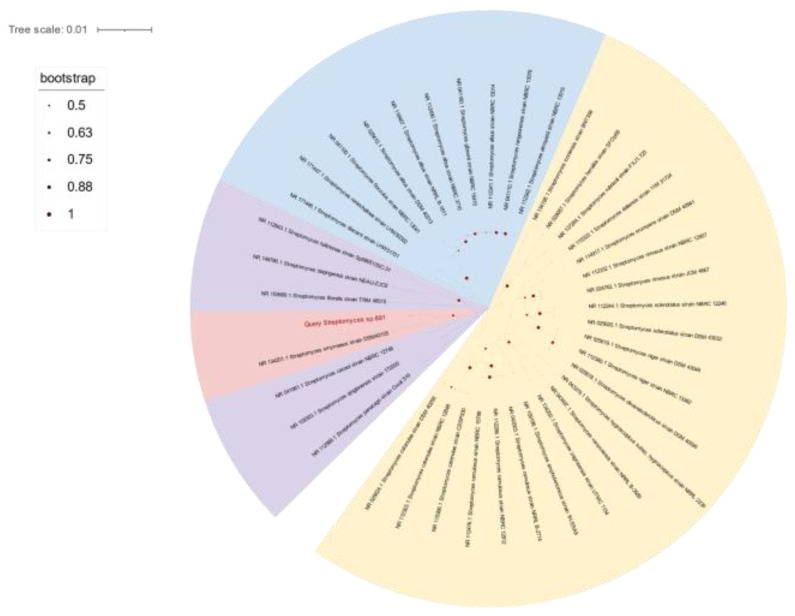
The 16*S* rRNA gene sequences-based phylogenetic tree of strain 891; all strains from the NCBI rRNA/ITS database.

**Figure 4:**
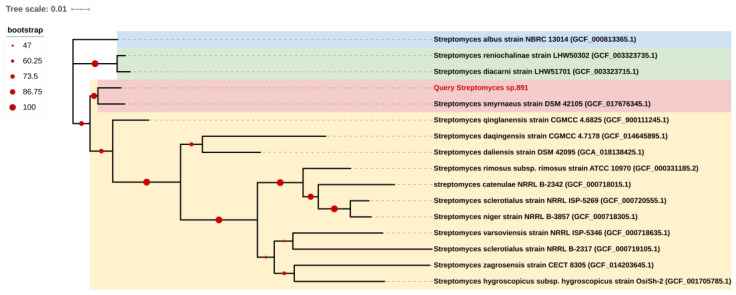
Whole genome sequence-based phylogenetic tree of strain 891; all strains from the NCBI Genome database.

The opaque colonies of strain 891 were spherical and wrinkled, and its curly or spiral mycelia cultivated for 14 days were differentiated into spore chains (Figure 2), suggesting this isolate had the common morphological characteristics of *Streptomyces*. BLAST analysis of the 16*S* rRNA gene sequence displayed that strain 891 had the highest similarity (98.93%) with the strain *Streptomyces smyrnaeus* DSM 42105. By hybridizing next-step generation (Illumina) and third-generation (PacBio) sequencing technologies, a high-quality chromosome-level genome of strain 891 together with a plasmid was assembled, and its sequence was deposited into GenBank and linked to BioProject PRJNA615006. Further, based on the 16*S* rRNA gene and whole genome sequences phylogenetic analysis, this strain made a monophyletic group with *S. smyrnaeus* DSM 42105, deposited in the NCBI database (Figure 3 and Figure 4) [18]. The average nucleotide identity (ANI) value for strains 891 and DSM 42105 was determined as 87.04%, indicating these strains belonged to species that were significantly different [19]. Accordingly, strain 891 was undoubtedly classified into the genus *Streptomyces*.

### 2.2. Genome Features of Strain 891

The whole genome of strain 891 consisted of one linear chromosome and a linear plasmid with 7,804,062 bp and 35,476 bp, respectively (Table 1). As many as 6871 protein-encoding genes were present in the chromosome and the longest genes had 78,261 bp. The GC content of the gene region was 71.44%, which was greater than that (68.87%) of the whole genome (Table S2). In strain 891, the total RNA pool included 18 rRNAs, 57 tRNAs, and 103 ncRNAs, in which the majority of these tRNAs were located in the chromosome’s intermediate region. It is normal that no rRNA, tRNA, or any non-coding RNA gene was present in the plasmid of strain 891. Essential genes associated with cell maintenance, including transcription, translation, and DNA replication, are usually sited in the “core” region of the chromosome of the *Streptomyces* genome [20,21]. The BLAST online alignment tool was used to identify regions of nucleotide similarity between the strain 891 complete plasmid sequence and the nr database maintained by NCBI database. The result showed no very similar plasmid sequence detected in other bacterial species or even genera. Fifteen predicted CRISPRs were present in strain 891, and two of them included more than 20 spacers located at the end of the linear chromosome (Table S3). The type I-B CRISPR-Cas immune system in strain 891 consists of seven CRISPR-associated (*cas*) genes flanked by two CRISPR loci (Table 2), which constitute its powerful immune system [22].

### 2.3. Genome Sequence Annotation of Strain 891

**Figure 5:**
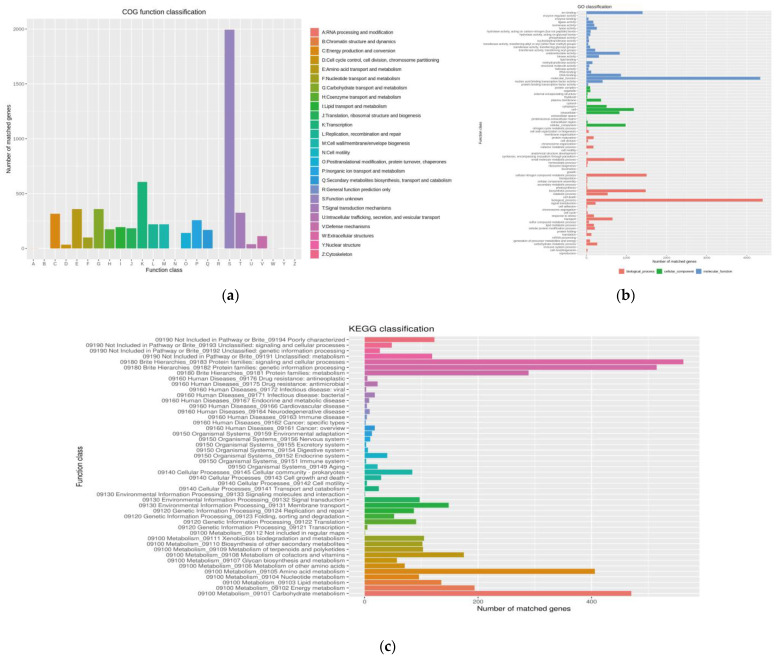
Functional gene annotation of strain 891: (**a**) Orthologous Groups of proteins (COG) analysis; (**b**) Gene Ontology (GO) analysis; (**c**) Kyoto Encyclopedia of Genes and Genomes (KEGG) analysis.

To predict protein sequences, 6871 non-redundant genes were subjected to similarity analysis based on five public databases. According to the COG database, the number of unknown function genes was the highest, and up to 1994, accounted for 29.02% of total protein-encoding genes. That was followed by “Transcription”, “Carbohydrate transport and metabolism”, and “Amino acid transport and metabolism” as the most gene-rich classes in the COG groupings (Figure 5). GO analysis was used to categorize genes into three categories according to matches with known sequences. The largest functional groups in the biological process category were cellular nitrogen compound metabolic process. In the cellular component category, the largest functional groups were cell, and the largest functional groups in the molecular function category were ion binding. Of the eight classifications of KEGG pathways, metabolism (carbohydrate metabolism and amino acid) contained the highest number of genes, followed by brite hierarchies (protein families: signaling and cellular processes, genetic information processing, metabolism) (Figure 5). These findings suggest the presence of an enriched and varied array of carbohydrates and amino metabolism functions that enable higher energy conversion efficiency.

### 2.4. Additional Annotation on Prophage, Genomic Islands, Antibiotic Resistance, and Carbohydrate Genes

**Figure 6:**
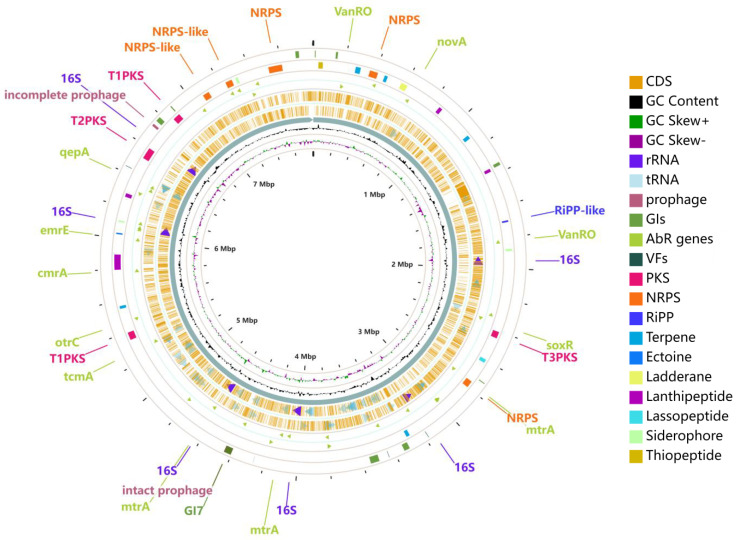
General chromosome features of strain 891. (From outside to inside, putative prophages, Genomics Islands, VFs; putative biosynthetic gene clusters; AbR genes on the forward strand; AbR genes on the reverse strand; putative protein-encoding sequences (CDSs) on the forward strand; CDSs on the reverse strand; and GC content, GC skew+, GC skew−).

Using the PHASTER (PHAge Search Tool Enhanced Release) approach, one complete prophage with a length of 45 kb and 50 genes and one truncated prophage with a size of 14 kb and 25 genes were found in the chromosome of strain 891 (Figure 6). A total of ten genomic islands (GIs) was predicted, in which GI7 overlapped with an intact prophage. Strain 891 harbored several significant pathogenic and virulence-related genes (Virulence Factors, VFs) including *KatAB*, *ClpC*, *IdeR*, *RelA*, and *Mycobactin*, which were potential targets for developing new treatment methods and therapies, as well as proposed functions shown in Table S5. The finding of 37 antibiotic resistance genes (AbR genes), 21 antibiotic target genes, and one antibiotic biosynthesis gene was made using the Antibiotic Resistance Database (CARD). These antibiotic resistance genes of strain 891 are marked as triangles between circles 3 and 4 in Figure 6, and several genes (such as *vanRO* and *mtrA*) were found to be two-component system response regulators. CAZy (Carbohydrate-Active Enzymes Database) comprises families of enzymes related to glycosidic bond degradation, modification, and generation and consist of various classes including glycoside hydrolases (GHs), critical enzymes for lignocellulosic biomass degradation, glycosyl transferases (GTs), polysaccharide lyases (PLs), carbohydrate esterases (CEs), auxiliary active enzymes (AAs), and carbohydrate-binding modules (CBMs) [23]. CAZy analysis of strain 891 revealed a total of 250 potential genes including 99 GHs, 62 GTs, 39 CEs, 23 CBMs, 21 AAs, and 6 PLs.

### 2.5. Analysis of Secondary Metabolite Biosynthetic Gene Clusters

**Figure 7:**
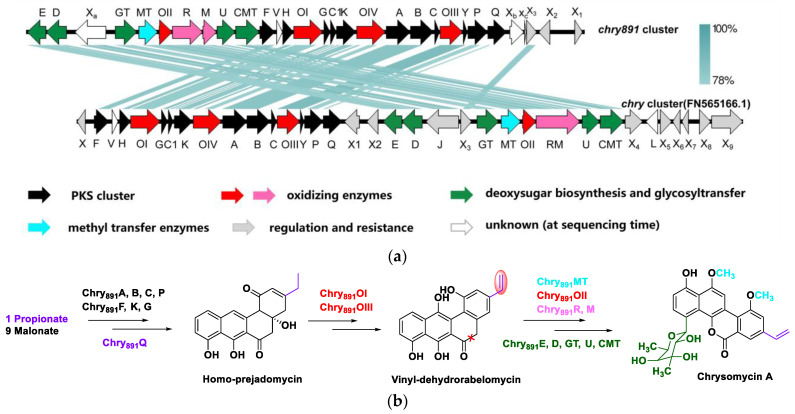
Comparison of chrysomycin BGC in strain 891 with that in strain FN566166.1 (**a**) and putative biosynthetic pathway for chrysomycin A in strain 891 (**b**).

AntiSMASH analysis resulted in discovery of 26 putative secondary metabolite BGCs including 6 PKSs, 6 NRPSs, 5 terpenes, and 9 other unknown clusters in strain 891 (Table S4). Five of these BGCs showed high similarity (>80% of genes showed similarity) with those BGCs responsible for biosynthesis of geosmin, ectoine, desferrioxamine E, marineosin, and isorenieratene. It is noteworthy that cluster 21 displayed 74% similarity with reference chrysomycin BGC. The biosynthesis of gilvocarcins M and V consisted of two initial substrates (propionyl CoA and acetyl CoA) and nine malonyl CoAs as extending units as well as a series of successive oxidation, reduction, rearrangement, and methylation [9]. Owing to the same motif of chrysomycin and gilvocarcin, these natural products have similar biosynthetic pathways (Figure 7b). Considering the identity value, the BGC responsible for biosynthesis of chrysomycins was identified and consisted of 30 ORFs, which were respectively named based on their precedent *chry* genes (FN565166.1) from a cosmid library of *Streptomyces albaduncus* AD819 [24].

By further comparison with the characterized BGC for biosynthesis of chrysomycin in strain AD819 (Figure 7a), *chry891* had fewer regulatory or resistance mediating genes, such as absent *chryX* and *X*_4–9_. Sequence alignment analysis suggested that *chry891_R* and *chry891_M* were two separate ORFs owing to presence of a stop codon between them. Nearly all genes responsible for the PK scaffold and other genes accountable for the sugar moiety were positionally adjusted in the corresponding part of the reference *chry* cluster. Blastp predicted *chry891_X*_3_ was one member of the Multiple Antibiotic Resistance Regulator (MarR) family. MarR homologs govern stress responses, pathogenicity, and the breakdown or export of hazardous substances such as phenolic compounds, antibiotics, and common household detergents [25]. Gene *chry891_X*_2_ was putatively responsible for coding CitB, a DNA-binding response regulator with REC and HTH domains, which belongs to the NarL/FixJ family. CitB is a member of the two-component regulatory system CitA/CitB, which is required for expression of citrate-specific fermentation genes. Phosphorylated CitB binds to two sites in the citS–citC intergenic region, where it probably activates transcription of both genes [26]. Besides that, three unknown genes were integrated in the *chry891* cluster, and premised on their conserved domains, *chry891_X_a_* was most likely *emrB*, encoding a DHA2 family efflux MFS (major facilitator superfamily) transporter permease subunit. As the largest family of transporters, the MFS is a typical type of multidrug resistance efflux pump, associated closely with antibiotic resistance and taking part in several important processes of bacterial cell physiology, including cell to cell communication, and increasing the virulence potential of several bacterial pathogens [27]. *Chry891_X_b_* and *chry891_X_c_* were predicted to encode 4′-phosphopantetheinyl transferase superfamily protein and FAD-dependent monooxygenase, which are important co-factors of secondary metabolite biosynthesis catalytic enzymes. Compared to *chry* cluster (FN565166.1), *chry891* cluster retrenched at least seven genes involved in regulation or resistance and enlarged three candidates for regulatory or co-factors, which could further promote chrysomycin biosynthesis. In addition, the gene assemblies in charge of the PK scaffold and sugar moiety nearly swapped positions, which demonstrated the plasticity of horizontal gene transfer in type II PKS.

## 3. Conclusions

A high-quality chromosome-level genome and a plasmid were first de novo assembled for chrysomycin A-producing marine strain 891 by a combination of next-step generation (Illumina) and third-generation (PacBio) sequencing technologies. Phylogenetic analysis of the 16*S* rRNA gene and genome sequences revealed that this strain was undoubtedly classified into the *Streptomyces* genu*s*. Since the function of bacterial defense to self-toxicity is intimately linked to prophages, genomic islands, virulence factors, and antibiotic resistance genes, the abundance of the corresponding genes in strain 891 should significantly contribute to chrysomycin production capability in vivo without self-toxicity, as it is a high-yielding strain [28,29,30]. AntiSMASH analysis of this strain resulted in the discovery of one key BGC T2PKS responsible for the biosynthesis of chrysomycin, and its biosynthesis pathway was putatively proposed. These findings may pave the way for the full development of strain 891 to construct a robust strain with high-yield chrysomycin A production using genetic engineering techniques and combinatorial biology approaches.

## 4. Materials and Methods

### 4.1. Microbes and Cultivation

Strain 891 was isolated from mangrove sediments of the South China Sea in 2017, and a suspension of culture containing mycelia in ISP2 supplemented with glycerol (20% *v*/*v*) was stored at −80 °C. This strain was inoculated into the ISP2 medium to culture at 28 °C and cultivated for several days followed by morphology inspection and genome sequencing.

### 4.2. Phylogenetic Analysis

Strain 891 was identified based on phylogenetic analysis of 16*S* rRNA gene and genome sequences. The 16*S* rRNA gene phylogenetic tree was delineated via a neighbor-joining mode which was constructed using the Tamura3-parameter model in MEGA11 with 1000 bootstrap replicates [31]. We generated the genomic phylogenetic tree by using GTDB-TK [32] (Figure 4). In addition, an ANI value of the two strains was calculated by the online web-server EZBioCloud ANI tool (ANI Calculator, https://www.ezbiocloud.net/tools/ani) [33].

### 4.3. Genome Sequencing and Assembly

Genomic DNA was extracted following CTAB extraction protocol. The integrity and purity were assessed by 1% agarose gel electrophoresis and then dissolved in sterile water and adjusted to a concentration of 149 ng/μL. The next-generation and the third-generation sequencing technologies utilized the Illumina HiSeq 2500 platform (San Diego, CA, USA) and PacBio Sequel platform (Menlo Park, CA, USA), respectively. The next-generation sequencing was performed using TruSeqTM DNA Sample Prep Kit (Illumina) with Standard Illumina library preparation protocols. The libraries were quantified by Pico Green dsDNA quantitation assay and qualified by the Agilent Technologies 2100 bioanalyzer (Santa Clara, CA, USA). The third-generation genome sequencing libraries were performed using Pacbio Template Prep Kit 1.0 (Pacbio) with Standard 20 kb Template Preparation protocols (Using BluePippin Size Selection). The libraries were quantified by Qubit 3.0 Fluorometer (Woodlands Central, Singapore) after each bead purification procedure and qualified by the Agilent Technologies 2100 bioanalyzer. After sequencing, the paired-end raw data were saved in FASTQ format. The third-generation sequencing reads were assembled by HGAP (v4, http://www.pacb.com/devnet/) [34] and CANU (v1.7.1, https://canu.readthedocs.io/en/latest/) [35] softwares into contigs.

Quality control on paired-end raw reads from next-generation sequencing data used FastQC, the 3′ end of DNA adapter contamination was decontaminated with Adapter Removal protocol, and then SOAPdenovo2 was used to perform quality correction on all reads based on the k-mer frequency; the k-mer setting used for the correction was 17. Finally, Pilon software (v1.18, https://github.com/broadinstitute/pilon) was utilized to correct the third-generation contigs with the above-mentioned high-quality second-generation sequencing data and stitch together to assemble a complete strain 891 genome sequence [36]. GeneMarkS software (v4.32, http://topaz.gatech.edu/GeneMark/) was used to predict protein-coding genes in strain 891 genome [37]. The tRNA genes were predicted using tRNAscan-SE (v1.3.1, http://lowelab.ucsc.edu/tRNAscan-SE/) and rRNA genes using Barrnap (v0.9, http://www.vicbioinformatics.com/software.barrnap.shtml). CRISPRs were forecasted by obtaining directed repeats (DRs) and spacers in the whole genome [38].

### 4.4. Genome Sequence Annotation

All predicted protein-encoding genes were annotated using DIAMOND blastp to perform sequences alignment based on the NCBI NR database, COG database, and Swiss-Prot database, respectively [39]. Gene annotations of KEGG (Kyoto Encyclopedia of Genes and Genomes) Ortholog and Pathway was mainly completed by KEGG’s KAAS (v2.1, https://www.genome.jp/tools/kaas/) automated annotation system [40]. Genes were also annotated using Blast2GO software (v1.0, https://www.blast2go.com/) in Gene Ontology (GO) [41].

### 4.5. Additional Bioinformatics Analysis

PHASTER (PHAge Search Tool Enhanced Release, http://phaster.ca/) was used to predict the presence of prophages in the genome of strain 891 [42]. Predicting the presence of genomics islands was conducted through IslandViewer 4 [43]. The gene-coding protein sequence via BLAST software (v2.5.0, https://blast.ncbi.nlm.nih.gov/Blast.cgi) was compared with the amino acid sequence (Set A) in the Virulence Factors of Pathogenic Bacteria database to predict the presence of virulence factor-related genes and antibiotic resistance genes in the genome. Hmmscan software (v3.2.1, http://hmmer.org/) was used to predict the presence of CAZy enzyme genes in genomic sequence.

### 4.6. Analysis of Secondary Metabolite Biosynthetic Gene Clusters

Secondary metabolite BGCs of strain 891 were predicted by antiSMASH 6.0 (https://antismashdb.Secondarymetabolites.org/#/start, accessed on 7 March 2022) with relaxed detection, and 7 of 9 extra features were selected to obtain copious information for analysis [44].

## Figures and Tables

**Table 1:** Genomic features and annotation of strain 891.

Features	Chromosome	Plasmid
Genome topology	linear	linear
Genome size (bp)	7,804,062	35,476
GC content (%)	71.11	68.31
Open reading frames	6871	37
Gene total length (bp)	6,656,877	30,870
Gene density (genes per kb)	0.88	1.043
Longest gene length (bp)	78,261	4968
Gene average length (bp)	968.84	834.32
GC content in gene region (%)	71.44	68.62
rRNA genes	18	0
tRNA genes	57	0
ncRNA genes	103	0
Secondary metabolite BGCs	26	0
Genes assigned to Swiss-Prot	4236	5
Genes assigned to KEGG	2232	2
Genes assigned to GO	4675	7
Genes assigned to NR	6581	25
Genes assigned to COG	5808	11
Genes assigned to CARD	55	0
Genes assigned to CAZy	250	0
CRISPR repeats	15	0
GenBank accession number	CP050693	CP050694

**Table 2:** Location of CRISPR-associated genes and repeats in strain 891.

	Positions	Functions
CRISPR repeat No.1	7,701,174–7,702,715	CRISPR repeat sequences
*cas* gene	7,703,460–7,703,765	CRISPR-associated endoribonuclease Cas6
	7,705,207–7,706,280	type I-B CRISPR-associated protein Cas7/Cst2/DevR
	7,706,474–7,707,001	CRISPR-associated protein Cas5
	7,707,097–7,709,430	CRISPR-associated helicase/endonuclease Cas3
	7,709,427–7,709,942	CRISPR-associated protein Cas4
	7,709,942–7,710,922	CRISPR-associated protein Cas1
	7,711,064–7,711,192	CRISPR-associated protein Cas2
CRISPR repeat No.2	7,712,784–7,714,256	CRISPR repeat sequences

## Data Availability

The complete genome sequence data reported in this study are available within NCBI GenBank BioProject PRJNA615006.

## Supplementary Materials

The following supporting information can be downloaded at: https://www.mdpi.com/article/10.3390/md20050287/s1, Table S1: Information associated with the different chrysomycin (A)-producing species; Table S2: The statistical results of sequence-length distribution of the third-generation sequencing data; Table S3: CRISPR arrays in strain 891; Table S4: Putative biosynthetic gene clusters (BGCs) coding for secondary metabolites in strain 891; Table S5: Putative function of Virulence Factors in strain 891. References [45,46,47] are cited in the supplementary materials.
