# Supplementary material for: Comprehensive Genomic Analysis of Marine Strain Streptomyces sp. 891, an Excellent Producer of Chrysomycin A with Therapeutic Potential
Source: Mar Drugs. 2022 Apr 24;20(5):287. doi: 10.3390/md20050287 (PMC9144908; doi:10.3390/md20050287)
Supplement: Supplementary file 1 [file marinedrugs-20-00287-s001.zip › marinedrugs-1677005-supplementary.pdf]

Supplementary material

Comprehensive Genomic Analysis of One Marine Strain *Streptomyces* sp. 891, an Excellent Producer of Chrysomycin A with Therapeutic Potential

Xu Hu [1,2,†], Yuqi Tang [1,†], Yuanyuan Liu [1], Xinwei Pei [1], Ziwei Huang [1], Fuxing Song [3] and Huawei Zhang [1,*]

1 School of Pharmaceutical Sciences, Zhejiang University of Technology, Hangzhou 310014, China
2 College of Life and Environmental Sciences, Hangzhou Normal University, Hangzhou 311121, China
3 School of Light Industry, Beijing Technology and Business University, Beijing 100048, China
* Correspondence: hwzhang@zjut.edu.cn
† These authors contributed equally to this work.

Table S1. Information associated with the different chrysomycin (A)-producing species.

Strain	Source	Yield of chrysomycin A	Ref.
Streptomyces sp. A-419	Soil (New York Botanical Garden, USA)	5~10 mg/L	[1]
Streptomyces virens	Soil (Russia)	/	[2]
Streptomyces albaduncus C38291 (ATCC 14698)	Soil (Colombia)	/	[3]
Streptomyces albaduncus AD819	Soil (New York Botanical Garden, USA)	3 mg/L	[4,5]
Streptomyces sporoverrucosus strain CR1	Soil (Jammu Hill, India）	5 mg/L	[6]
Streptomyces sp.OA161	Mixed sample (India)	/	[7]
Streptomyces sp. 891	Sediment (South China Sea)	3,600 mg/L	[8]

Table S2. The statistical results of sequence-length distribution of the third-generation sequencing data of strain 891.

Sample Name	Sequence
Total Number (#)	187,835
Total Length (bp)	1,461,334,907
Max Length (bp)	50,211
Min Length (bp)	200
N20 (bp)	12,425
N50 (bp)	9,471
N90 (bp)	5,502
Sequences Number (≥1kb)	180,244
GC content %	68.87

Table S3. CRISPR arrays in strain 891.

No.	Start	End	Number of Spacer	Length (bp)	Genome (%)
1	7,701,174	7,702,715	23	1,541	0.0197
2	7,712,784	7,714,256	22	1,472	0.0189

3	3,190	3,397	3	207	0.0027
4	1,539,060	1,539,188	1	128	0.0016
5	1,796,106	1,796,192	1	86	0.0011
6	2,722,200	2,722,317	1	117	0.0015
7	2,734,535	2,734,645	1	110	0.0014
8	3,081,958	3,082,182	1	224	0.0029
9	3,235,213	3,235,352	1	139	0.0018
10	4,248,405	4,248,516	1	111	0.0014
11	5,500,464	5,500,577	1	113	0.0014
12	5,508,296	5,508,404	1	108	0.0014
13	5,661,901	5,662,010	1	109	0.0014
14	6,161,260	6,161,392	1	132	0.0017
15	6,347,661	6,347,778	1	117	0.0015

Table S4. Putative biosynthetic gene clusters (BGCs) coding for secondary metabolites in strain 891.

BGC	Core potentials	From	To	Most similar known cluster (%)
Cluster 1	thiopeptide, LAP	32,672	60,344	-
Cluster 2	Terpene, RiPP	268,772	300,586	citrulassin E (66%)
Cluster 3	NRPS, NRPS-like	359,628	412,242	enduracidin (8%)
Cluster 4	Terpene	457,490	477,651	-
Cluster 5	ladderane, Other	571,337	612,704	spiroindimicin/indimicin/lynamicin (6%)
Cluster 6	lanthipeptide	853,073	875,787	SapB (75%)
Cluster 7	Terpene	1,103,217	1,126,499	hopene (61%)
Cluster 8	lanthipeptide	1,352,217	1,371,434	-
Cluster 9	RiPP-like	1,693,792	1,704,652	-
Cluster 10	siderophore	1,873,704	1,887,891	-
Cluster 11	T3PKS, NRP	2,405,467	2,446,510	echoside (35%)
Cluster 12	lassopeptide, RiPP	2,595,968	2,618,593	aborycin (50%)
Cluster 13	NRPS, Other	2,758,394	2,801,884	2'-chloropentostatin / 2'-amino-2'-deoxyadenosine (6%)
Cluster 14	Terpene	3,273,143	3,295,228	isorenieratene (85%)
Cluster 15	T1PKS, butyrolactone	5,346,521	5,395,065	reveromycin A (15%)
Cluster 16	Terpene	5,550,835	5,568,424	geosmin (100%)
Cluster 17	lanthipeptide, Polyketide	5,797,742	5,894,239	xantholipin (61%)
Cluster 18	Ectoine	6,022,782	6,031,572	ectoine (100%)
Cluster 19	siderophore, Other	6,100,486	6,110,067	desferrioxamine E (100%)
Cluster 20	lanthipeptide	6,262,668	6,285,460	-
Cluster 21	T2PKS, Polyketide	6,529,403	6,601,876	chrysomycin (74%)
Cluster 22	T1PKS, Polyketide	6,837,722	6,884,576	marineosin (100%)
Cluster 23	NRPS-like	7,073,517	7,115,564	echoside (11%)
Cluster 24	NRPS-like, Saccharide	7,235,261	7,278,035	paromomycin (5%)
Cluster 25	siderophore, Polyketide	7,302,042	7,318,494	macrotetrolide (33%)
Cluster 26	NRPS	7,519,764	7,606,851	marformycin (8%)

Table S5. Putative function of Virulence Factors in strain 891.

VFs	VF_id	VF_Funcation
KatA	VFG001861	1. A periplasmic catalase, expressed maximally during the post-exponential phase.<br>2. Important for intracellular survival and transmission.
ClpC	VFG000079	1. An ATPase promoting early escape form the phagosome of macrophages.<br>2. ClpC is also required for adhesion and invasion, possibly by modulating the expression of InlA,InlB and ActA.
IdeR	VFG001406	Repressor of iron acquisition genes, activator of iron storage genes and a positive regulator of oxidative stress responses.
RelA	VFG001826	Associated with stationary phase adaptation and long-term survival.
Mycobactin	VFG001818	Cell-association mycobactin participates in iron internalization and/or to serve as a temporary iron-holding molecule to prevent sudden influx of excess iron if the metal suddenly becomes available after a period of iron limitation.

Reference

1. Strelitz, F.; Flon, H.; Asheshov, I.N. Chrysomycin: A New Antibiotic Substance for Bacterial Viruses. *J. Bacteriol.* **1955**, *69*, 280–283, doi:10.1128/jb.69.3.280-283.1955.
2. Gauze, G.F.; Sveshnikova, M.A.; Maksimova, T.S.; Olʹkhovatova, O.L.; Bazhanov, V.S. Formation of New Antibiotic, Virenomycin, by a Culture of *Streptomyces virens* sp. nev. *Antibiotiki* **1977**, *22*, 963–967.
3. Matson, J.A.; Rose, W.C.; Bush, J.A.; Myllymaki, R.; Bradner, W.T.; Doyle, T.W. Antitumor Activity of Chrysomycins M and V. *J. Antibiot.* **1989**, *42*, 1446–1448, doi:10.7164/antibiotics.42.1446.
4. Carter, G.T.; Fantini, A.; James, J.C.; Borders, D.B.; White, R.J. Biosynthesis of Chrysomycins A and B Origin of the Chromophore. *J. Antibiot.* **1985**, *38*, 242–248, doi:10.7164/antibiotics.38.242.
5. Kharel, M.K.; Nybo, S.E.; Shepherd, M.D.; Rohr, J. Cloning and Characterization of the Ravidomycin and Chrysomycin Biosynthetic Gene Clusters. *ChemBioChem* **2010**, *11*, 523–532, doi:10.1002/cbic.200900673.
6. Jain, S.K.; Pathania, A.S.; Parshad, R.; Raina, C.; Ali, A.; Gupta, A.P.; Kushwaha, M.; Aravinda, S.; Bhushan, S.; Bharate, S.B.; et al. Chrysomycins A–C, Antileukemic Naphthocoumarins from *Streptomyces Sporoverrucosus*. *RSC Adv.* **2013**, *3*, 21046, doi:10.1039/c3ra42884b.
7. Muralikrishnan, B.; Dan, V.M.; Vinodh, J.S.; Jamsheena, V.; Ramachandran, R.; Thomas, S.; Dastager, S.G.; Kumar, K.S.; Lankalapalli, R.S.; Kumar, R.A. Anti-Microbial Activity of Chrysomycin A Produced by *Streptomyces* sp. against *Mycobacterium Tuberculosis*. *RSC Adv.* **2017**, *7*, 36335–36339, doi:10.1039/C7RA05576E.
8. Ni, H.-J.; Lv, S.-Y.; Sheng, Y.-T.; Wang, H.; Chu, X.-H.; Zhang, H.-W. Optimization of Fermentation Conditions and Medium Compositions for the Production of Chrysomycin A by a Marine-Derived Strain *Streptomyces* sp. 891. *Prep. Biochem. Biotechnol.* **2021**, *51*, 998–1003, doi:10.1080/10826068.2021.1885046.
